# Sirolimus enhances the protection achieved by a DNA vaccine against *Leishmania infantum*

**DOI:** 10.1186/s13071-020-04165-4

**Published:** 2020-06-09

**Authors:** Alba Martínez-Flórez, Clara Martori, Paula L. Monteagudo, Fernando Rodriguez, Jordi Alberola, Alhelí Rodríguez-Cortés

**Affiliations:** 1 Departament de Farmacologia, de Terapèutica i de Toxicologia, Universitat Autònoma de Barcelona, Bellaterra, Barcelona, Spain; 2 Centre de Recerca En Sanitat Animal (CReSA), Institut de Recerca i Tecnologia Agroalimentàries (IRTA), Campus UAB, Bellaterra, 08193 Barcelona, Spain; 3 Department of Microbiology, Icahn School of Medicine at Mount Sinai, New York, NY 10029 USA

**Keywords:** *Leishmania*, Vaccine, Visceral leishmaniasis, Hamster, Sirolimus, mTOR, DNA vaccine

## Abstract

**Background:**

Leishmaniases are a group of neglected tropical parasitic diseases, mainly affecting vulnerable populations of countries with poor socioeconomic status. Development of efficient vaccines is a priority due to the increasing incidence of drug resistance and toxicity to current treatments. In the search for a safe and efficient protective vaccine for human and dog visceral leishmaniases, we analyzed the suitability of the immunomodulatory drug sirolimus (SIR) to boost a preventive DNA vaccine against leishmaniasis. SIR is an already marketed drug that has been described to boost immune protection against different disease models and has also emerged as a promising therapeutic drug against *L. major*.

**Methods:**

Syrian hamsters were treated with SIR concomitantly with the administration of a DNA vaccine formulation consisting in four plasmids carrying the *Leishmania* genes *LACK*, *TRYP*, *PAPLE22* and *KMPII*, respectively. Two weeks after the last vaccination, the animals were infected intraperitoneally with *L. infantum* parasites. Five weeks post-infection the parasite load was measured by real-time PCR in target tissues and immune response was evaluated by determining anti-*Leishmania* specific antibodies in combination with cytokine expression in the spleen.

**Results:**

Our results show that the DNA vaccine itself efficiently reduced the burden of parasites in the skin (*P* = 0.0004) and lymph nodes (*P* = 0.0452). SIR administration also enhanced the protection by reducing the parasite load in the spleen (*P* = 0.0004). Vaccinated animals with or without SIR co-treatment showed lower IFN-γ expression levels than those found in the spleen of control animals. mRNA expression levels of NOS2 and IL-10 were found to be significantly higher in the vaccinated plus SIR treated group.

**Conclusions:**

Co-administration of SIR enhances a DNA vaccination regimen against *L. infantum*, improving the reduction of parasite load in skin, lymph node and spleen. The analysis of immune markers in the spleen after challenge suggests that the trend to recover naïve levels of IFN-γ and IL-10, and the concurrent higher expression of NOS2, may be responsible for the protection induced by our vaccine co-administered with SIR.
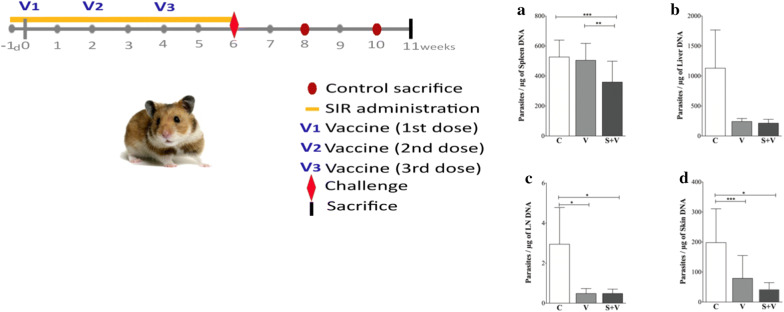

## Background

Leishmaniasis is a neglected disease caused by protozoan parasites belonging to the genus *Leishmania*. Transmitted by infected female sand fly vectors, *Leishmania* spp. are responsible for over 1,500,000 human cases, resulting in more than 20,000 deaths per year, with the highest impact on the poorest people in developing countries [1]. Visceral leishmaniasis (VL), also known as kala-azar, is the most severe clinical form, resulting in a fatal outcome if left untreated [2]. *Leishmania infantum* is the causative agent of zoonotic VL in the Neotropical zone and in the Mediterranean Basin, with dogs playing an important role as peridomestic reservoirs and increasing the risk of infection of susceptible human populations co-habiting in the same area [3].

Protective immunity against *Leishmania* is mediated by both innate and adaptive immune responses. Thus, today we know that the effective activation of macrophages, dendritic cells (DCs), and antigen-specific CD4+ and CD8+ T cells are all needed to control the spread of *Leishmania* [4]. The immune response induced by *Leishmania* has been fully described in the mouse model, with a CD4+ Th1 response (IFN-γ and TNF-α secretion) leading to the resolution of the disease and protection, and a CD4+ Th2 responses (IL-10 and IL-4) being associated with disease pathology [5]. CD4+ Th1 cells activate CD8+ T cells which are required for optimal resistance, providing beneficial cytokines such as IFN-γ and specifically killing parasite-infected cells [6, 7]. Unlike experimental VL in mice, most infected people develop asymptomatic infections, which correlates with the activation of specific cell-mediated immunity and a Th1-proinflammatory immune response [8]. The active disease, however, has been linked to high antibody levels and a progressive Th2-deactivating immune response in the presence of a strong inflammatory reaction [9].

In spite of several efficient drugs currently available, in many occasions these drugs are of limited access to the affected population. On the other hand, the increasing incidence of toxicity cases and drug-resistant leishmaniasis, require the development of safe and efficient preventive vaccines [10, 11]. Currently, there are three licensed and commercialized vaccines for canine leishmaniases prevention, CaniLeish^®^, Leish-Tech^®^ and Letifend^®^ [12–14]. Despite several efforts made using experimental models [15], there is no licensed vaccine against human leishmaniasis yet.

DNA vaccines are a suitable approach for several reasons, including the ability of plasmid DNA to stimulate Th1 responses by the presence of CpG motifs [16]. In addition, DNA vaccines are safe, have lower production costs, and are easy to store because a cold-chain is not required [17]. Several DNA vaccination approaches against *Leishmania* have been developed to date, reaching different degrees of protection in mice but achieving less immunogenicity in more complex models [18]. Thus, the co-administration of immunomodulatory compounds may be of paramount importance to boost the immune response induced by DNA vaccines. For this purpose, many adjuvants have been used in different *Leishmania*-vaccine trials [19], but physicochemical incompatibilities and undesirable adverse reactions remain a major problem (reviewed in [20]).

Sirolimus (SIR), also known as rapamycin, is an immunosuppressant drug which is licensed and marketed primarily to prevent organ transplant rejection [21, 22]. SIR acts by binding and constraining the activity of the mechanistic (mammalian) target of rapamycin (mTOR), a highly conserved pathway among eukaryotic organisms that orchestrates cell adaptations to environmental changes by controlling cell survival strategies, cell proliferation and metabolic adaptations at the transcriptional and translational levels [23]. Recent studies have opened a whole new field of research by showing that SIR administration during the expansion and contraction phase of a primary immune response results in an increased magnitude and quality of CD8+ T memory cells [24]. SIR administration has shown promising adjuvant results on different vaccine trials against viruses [25, 26], cancer models [27, 28] and even against intracellular mycobacteria infection [29]. The protection achieved using SIR in these studies of vaccination was mediated by the generation of memory effector CD8+ T and the concurrent increase in IFN-γ production. In regards to *Leishmania*, a recent study using SIR as a therapeutic agent on a mouse model of cutaneous leishmaniasis showed promising results, pointing that this molecule could be a potential candidate for further research in the field of leishmaniasis [30].

We have previously studied the effect of a DNA vaccine carrying the *Leishmania* genes *LACK*, *TRYP*, *PAPLE22* and *KMPII* in a hamster model of leishmaniasis. When administered alone, it was poorly immunogenic but induced partial protection when followed by a protein vaccine boost [31]. The aim of the present study was to test in hamsters if the co-administration of SIR would enhance the protection afforded by a *L. infantum* DNA vaccine.

## Methods

### Parasites

The *L. infantum* strain MCAN/ES/92/BCN83 (zymodeme MON-1) was kindly provided by Dr Montserrat Portús, Universitat de Barcelona, Spain. It was obtained from a naturally infected and untreated dog and maintained through hamster passages to retain its full virulence. Hamsters were intraperitoneally infected with stationary promastigotes, and at 3 months post-infection, parasites were isolated from spleen samples incubated in R15 medium (RPMI 1640 medium (Gibco, Billings, USA) supplemented with 15% heat-inactivated fetal calf serum (Gibco), 1 M 2% HEPES (Gibco) and 1% of total volume of 10,000 U/ml penicillin, and 10,000 μg/ml streptomycin (Gibco)).

Promastigotes were maintained at 26 °C in R15 medium, and weekly passages were performed. A second passage of *Leishmania infantum* cultures at stationary growth (6-day culture) was used for infection to assure than > 85% of promastigotes were metacyclic and that virulence was retained [32]. Stationary promastigotes for infection were washed and resuspended in PBS at 1 × 10^7^ parasites/ml.

### Animals

Thirty-four male, 7-week-old, Syrian golden hamsters (*Mesocricetus auratus*, strain RjHAN-AURA) were obtained from Centre d’Elevage René Janvier (Le Genest-Saint-Isle, France). The animals were kept individually in plastic microfilter cages under BSL2 conditions at the Servei d’Estabulari of Universitat Autònoma de Barcelona (Barcelona, Spain), and food and water were provided *ad libitum*. The study comprised a large experiment with 30 hamsters randomly distributed into 3 experimental groups of 10 animals each: treated with SIR and DNA vaccinated group (S + V); DNA-vaccinated group (V); and PBS-inoculated control group (C). The 30 hamsters were further infected with *Leishmania infantum* (see below) and four additional hamsters were used as a non-infected control group (NI).

### Sirolimus treatment

Rapamune^®^ (sirolimus 1 mg/ml) was purchased from Pfizer (New York, USA) and kept under conditions specified by the manufacturer. Treatment doses of 0.075 mg/kg in 500 μl of PBS were prepared daily under aseptic conditions [24]. The S + V group received a daily intraperitoneal dose from day − 1 (one day prior to the first vaccination) for 6 consecutive weeks. The V and C groups of hamsters were handled under the same conditions and were PBS-inoculated.

### Immunization

The open reading frames (ORF) from *L. infantum* genes *TRYP* (GenBank: AF044679), *PAPLE22* (GenBank: AF123892), *LACK* (GenBank: U49695) and *KMPII* (GenBank: X95267) were individually cloned into a pVAX 1 vector (Invitrogen, Waltham, USA) as described elsewhere [18, 31]. Each DNA construction was purified using EndoFree Plasmid Maxi Kit (Qiagen, Venlo, Netherlands) according to the manufacturer’s instructions. The immunogenicity of these plasmids was previously tested [31].

Each hamster received three doses (2 weeks apart) of 100 µg of each plasmid administered intramuscularly into their hind limb (V and S + V groups). To maintain the same handling conditions, the C group was injected with physiological saline solution.

### Infection

Two weeks after the administration of the last vaccine dose, all animals but the NI group were intraperitoneally inoculated with 1 ml of PBS containing 1 × 10^7^*L. infantum* metacyclic promastigotes. Animal weight and typical VL clinical signs in the rodent model, i.e. hair and weight loss, and cutaneous lesions, were weekly evaluated.

### Necropsy and sample collection

**Fig. 1:**
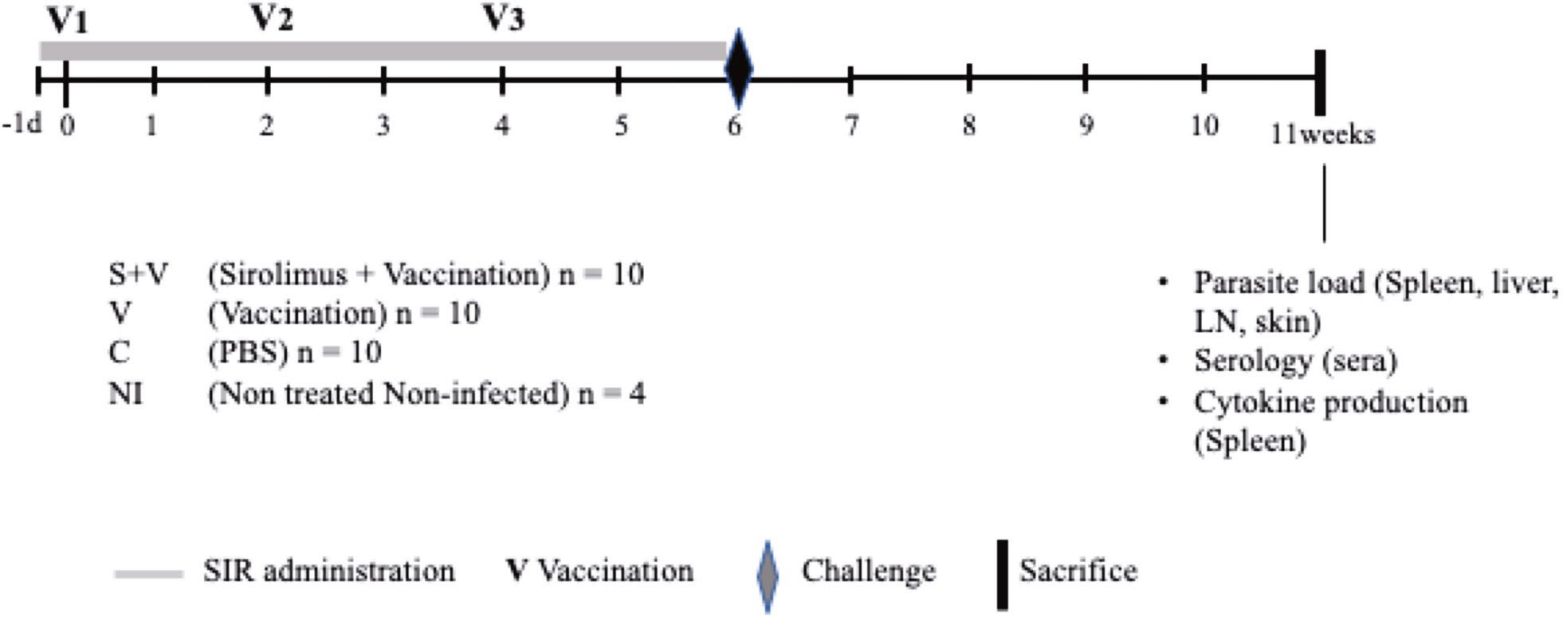
Graphical representation of the experimental assay. The study comprised a large experiment with a total of 34 Syrian golden hamsters randomly distributed in 4 groups: NI (non-infected group, *n* = 4), C (PBS-inoculated control group, *n* = 10), V (DNA vaccinated group, *n* = 10), and S + V (sirolimus-treated and DNA vaccinated group, *n* = 10). Hamsters received sirolimus or a PBS solution daily from one day prior to the first vaccination for 6 consecutive weeks. Three vaccine doses were administered, leaving a 2-week period between them. C, V and S + V animals were challenged two weeks after receiving the last vaccination dose and all hamsters were sacrificed 5 weeks later

All animals (*n* = 34) were euthanized at 5 weeks post-challenge (Fig. 1). Hamsters were anesthetized with a 100 μl intramuscular injection containing ketamine (42.8 mg/kg), acepromazine (1.4 mg/kg) and xylazine (9.5 mg/kg). An intracardiac puncture was performed to obtain 5 ml of blood sample from each animal, and the animals were then euthanized in a CO_2_ chamber. Samples of spleen, liver, submandibular lymph nodes (LN) and healthy pinnae skin from each hamster were aseptically obtained and kept on ice to avoid tissue degradation.

### Evaluation of humoral immune response: total hamster IgG anti-*L. infantum*

Blood samples were collected at the end of the study in hematology crystal tubes (BD Vacutainer^®^; BD, New Jersey, USA) and centrifuged at 2000×*g* for 15 min to obtain serum samples and were frozen until analyzed. ELISA plates (Costar high binding transparent flat bottom; Fisher Scientific, Waltham, USA) were coated overnight at 4 °C with 2 µg per well of Crude Total *Leishmania* Antigen (CTLA) in 100 µl of carbonate-bicarbonate coating buffer (0.1 M NaCO_3_-H_2_CO_3_ pH 9.6). CTLA was provided by Dr Cristina Riera, Universitat de Barcelona, Spain. Samples were diluted 1:400 in PBS with 0.05% Tween 20 and 1% skimmed milk powder (PBSTM) and serially diluted in the pre-coated plate. Plates were incubated at 37 °C in a humid atmosphere for 1 h and were then washed 3 times with PBST and once with PBS. Plates were then incubated for 1 h at 37 °C with goat anti-hamster IgG-HRPO (Bio-Rad, Hercules, USA) diluted 1:1000 in PBSTM. Antibody excess was removed with another cycle of washes as described above. Then, 100 µl of the peroxidase substrate tetramethylbenzidine (TMB) (Sigma-Aldrich, St. Louis, USA) was added to each well, and the plates were left to develop for 5 min. The reaction was stopped by the addition of 50 µl of 1 M H_2_SO_4_, and the absorbance was recorded at 450 nm (Anthos 2001 reader; Anthos Labtech instruments, Wals, Austria). The cut-off value was set as the average optical densities (ODs) of NI serum samples plus 3 times the standard deviation (cut-off = 0.015 OD).

### DNA extraction and real-time PCR absolute parasite quantification

The efficacy of the vaccination strategies was evaluated by measuring the parasite reduction in target organs [33, 34]. Total genomic DNA was obtained from 50 mg of spleen, liver, skin and LN samples of the study animals by using the High Pure Template Preparation Kit (Roche, Basel, Switzerland) following the mouse tail protocol provided in the manufacturer’s guidelines. DNA concentration was measured using a NanoDrop 2000 Spectrophotometer (Isogen Life Science, De Meern, The Netherlands). All DNA samples were kept at − 20 °C until the qPCR was performed.

Primers targeting conserved DNA regions of the kinetoplast minicircle DNA from *L. infantum* and TaqMan-MGB probes were used as formerly described [35] (Table 1). Triplicates of 25 ng of DNA of each sample, a negative control using nuclease-free water (Sigma-Aldrich) instead of DNA, and a standard curve were run in an Applied Biosystems StepOnePlus™ PCR instrument (Applied Biosystems, Waltham, USA) following a thermal cycling profile of 50 °C for 2 min, 95 °C for 10 min, 40 cycles at 95 °C for 15 s, and 60 °C for 1 min. The resulting data were analyzed using StepOnePlus™ software v2.3 (Applied Biosystems). The number of parasites per µg of DNA was calculated by interpolation to a standard curve generated by using a 10-fold serial dilution from 10^3^ to 10^−3^ promastigotes of a *L. infantum* culture (y = − 3.38x + 28.43). The median qPCR efficiency (99%), and slope (− 3.38) were calculated from three different replicates. Parasite quantification was linear between 10^3^ and 10^−2^ parasites per sample (*R*^2^ = 0.993).

**Table 1:** Sequence of primers and probe used for parasite detection [35]

Gene	Primer sequence (5ʹ-3ʹ)	
Kinetoplastid minicircle DNA	F	AACTTTTCTGGTCCTCCGGGTAG
	R	ACCCCCAGTTTCCCGCC
	P	FAM–AAAAATGGGTGCAGAAAT–MGB

*Abbreviations*: F, forward primer; R, reverse primer; P, probe

### Cytokine determination

Cytokine expression of all groups (*n* = 34) was studied in spleen tissue. Samples maintained in RNAlater (Ambion, Austin, USA) were homogenized with TRI Reagent (Ambion) and kept frozen until RNA extraction. Total RNA was extracted by using the RiboPure kit (Ambion) following the manufacturer’s instructions, and samples were then treated with DNases TURBO-DNA free kit (Ambion). Clean RNA samples were stored at − 80 °C until analyzed. Retrotranscription was carried out by using the High-Capacity cDNA Reverse Transcription kit (Applied Biosystems) following a thermal profile of 25 °C for 10 min, 37 °C for 120 min, and 95 °C for 5 min. Levels of IFN-γ, FoxP3, TNF-α, IL-10, IL-21, NOS2 and TGF-β expression were determined in addition to *RPL18* as a control gene to calculate relative gene expression levels [36]. To ensure amplification of cDNA sequences derived from the retrotranscription of the mRNAs of interest, primers were designed including exon boundary sequences based on basic primer information extracted and modified from Zivcec et al. [36] and Espitia et al. [37] (Table 2). Rat (*Rattus norvergicus*) sequences were used for exon-boundary area determination, as hamster sequences are not available for ENSEMBL analysis [38], and previous BLAST and CLUSTALW analyses confirmed sequence identity. Amplification of each sample was carried out in triplicate using SYBR select RT-PCR Master Mix Reagents (Applied Biosystems) with the aid of an Applied Biosystems StepOnePlus™ PCR instrument and StepOnePlus™ Software v2.3 (Applied Biosystems). The thermal cycling profile was 10 min at 50 °C, followed by 40 cycles of 95 °C for 10 min, 95 °C for 15 s, and 60 °C for 1 min. Melting curves assessed the specificity of our amplification products. Cytokine mRNA expression levels were normalized to the expression levels of the housekeeping gene *RPL18*. Fold induction levels were calculated by using the median values of the C group.

**Table 2:** Sequences of primers used for expressed cytokines detection

Gene	Primer sequence (5ʹ-3ʹ)	
RPL18	F	GTCCCTGTCCCGGATGATC
	R	GACAGTCCCCACAACCACG
IFN-γ	F	CTATGTCTGGCTGCTACTGCCA
	R	TTCACGACATCTAAGCTACTTGAGTTAA
TNF-α	F	CCAGACACTCACACTCAGATCATCTT
	R	CACTTGGTGGTTTGCTACAACGT
TGF-β	F	CTACCACGCCAACTTCTGTCTG
	R	TGTAGAGGGCAAGGACCTTACTG
FoxP3	F	CAAGTGGCCTGGTTGTGAGA
	R	TGATCTGCTTGGCAGTGCT’
IL-21	F	CCAGCTCCACAAGATGTAAAGGA
	R	GCTGACTTGAGTTTGGCCTTCT
IL-10	F	CATCGATTTCTCCCCTGTGAA
	R	GACGCCTTTCTCTTGGAGCTT
NOS2	F	CCAGAACAGAGGGCTCAAAGG
	R	CCTGCATCTCTTCCCGATAGAG

*Note*: Data extracted and modified from Espitia et al. [37] and Zivcec et al. [36]

*Abbreviations*: F, forward primer; R, reverse primer

### Statistical analysis

Exploratory data analysis and inference were carried out by using R statistical software v3.2.5 [39]. Differences among groups were analyzed using robust one-way ANOVA with corresponding pairwise *post-hoc* tests included in the *WRS2* package v0.4-0 [40, 41]. The results were considered statistically significant when *P* ≤ 0.05.

## Results

### Parasitological protection

Animals from group V showed a reduction in the number of parasites compared to group C in the skin (mean reduction of 60%; $\hat{\uppsi }$ = 94.14, 95% Confidence Interval (CI): 36.41–180.64, *P* = 0.0004) and LN (mean reduction of 83%; $\hat{\uppsi }$ = 1.04, 95% CI: 0.13–2.05, *P* = 0.0452). Non-significant differences were observed in the liver (mean reduction of 78%; $\hat{\uppsi }$ = 886.28, 95% CI: − 6.26–1902.01, *P* = 0.1072) and spleen (mean reduction of 4%; $\hat{\uppsi }$ = 48.41, 95% CI: − 222.59–286.74, *P* = 0.8372).

**Fig. 2:**
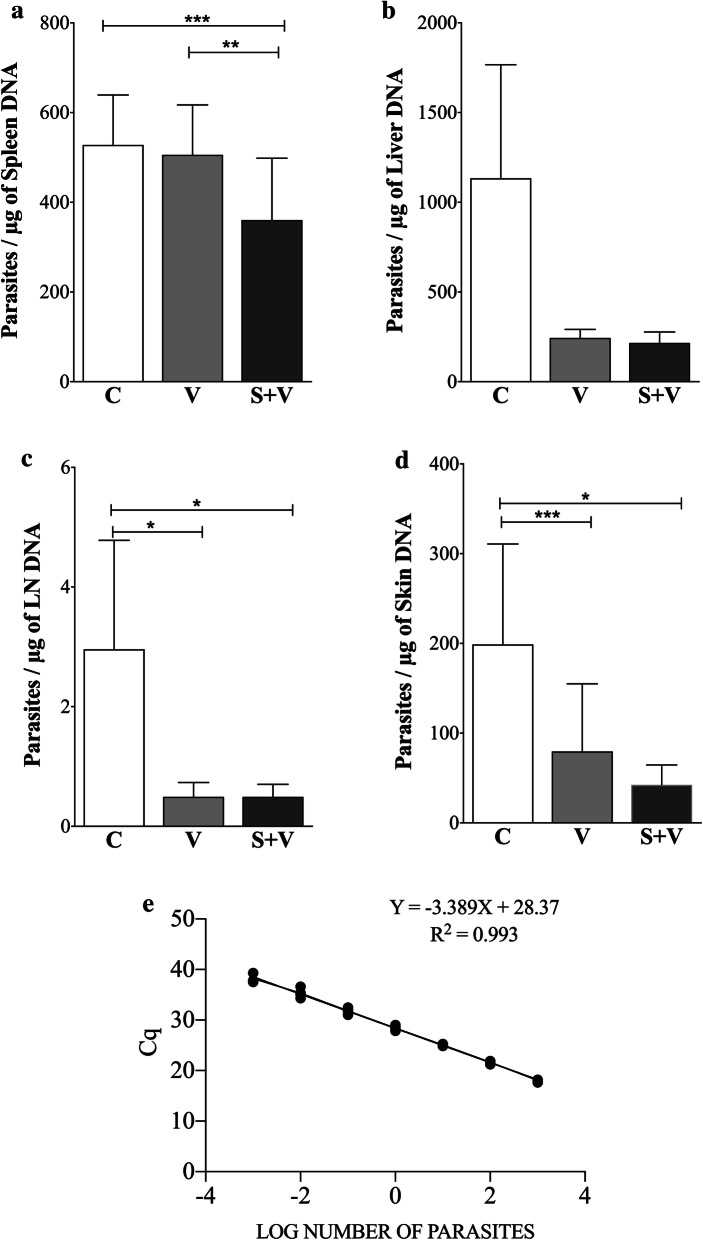
*Leishmania infantum* DNA load. qPCR analyses showing the number of parasites detected per μg of DNA extracted from the spleen (**a**), liver (**b**), lymph node (**c**) and skin (**d**) comparing the three *Leishmania*-infected studied groups: C (PBS-inoculated control group, *n* =10), V (DNA vaccinated group, *n* = 10), and S **+** V (sirolimus-treated and DNA vaccinated group, *n* = 10). Parasite number was calculated by interpolation to a standard curve constructed with a *L. infantum* promastigote culture (**e**). *Abbreviations*: Cq, quantification cycle. ANOVA; *P < 0.05, **P < 0.01, ***P < 0.001

Compared to group C, animals in the S + V group also had reduced numbers of parasites in skin (mean reduction of 79%; $\hat{\uppsi }$ = 80.75, 95% CI: 11.03–152.33, *P* = 0.0400) and LN (mean reduction of 83%; $\hat{\uppsi }$ = 0.95, 95% CI: 0.09–2.03, *P* = 0.0489), together with a significant protection in the spleen (mean reduction of 32%; $\hat{\uppsi }$ = 369.79, 95% CI: 180.09–561.06, *P* = 0.0004), with parasite burdens concurrently lower than group V (mean reduction of 57%; $\hat{\uppsi }$ = 321.37, 95% CI: 125.36–551.69, *P* = 0.0080). Although non-statistically significant ($\hat{\uppsi }$ = 936.50, 95% CI: 44.63–1945.62, *P* = 0.0598), there was also a noticeable trend of burden reduction in the livers of hamsters of the S + V group compared to group C (mean reduction of 81%) (Fig. 2).

### Analysis of the immune response

#### Anti-Leishmania IgG production

**Fig. 3:**
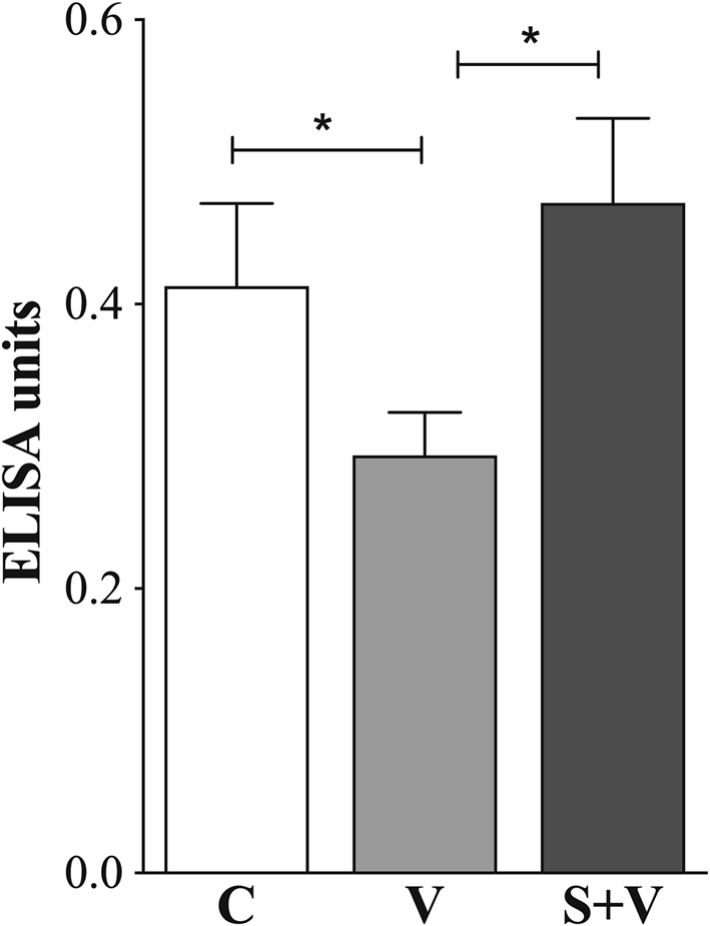
Comparison of anti-*Leishmania* IgG levels in group C (PBS-inoculated control group), V (DNA vaccinated group), and S + V (sirolimus-treated and DNA vaccinated group) at 5 weeks post-challenge. Antibody levels were measured by ELISA against crude total *L. infantum* antigen (*n* = 30). Cut-off value (mean + 3 SD) was set at 0.015 OD. ANOVA; * *P* < 0.05

All infected hamsters seroconverted and had IgG antibodies against CTLA at the time of sacrifice, 5 weeks post-infection (IgG cut-off = 0.015 OD). *Leishmania*-specific IgG production was reduced in group V animals when compared to group C ($\hat{\uppsi }$ = 0.12, 95% CI: 0.02–0.22, *P* = 0.0475), and S + V ($\hat{\uppsi }$ = − 0.12, 95% CI: − 0.22–0.04, *P* = 0.0180). In contrast, similar levels of *Leishmania*-specific antibodies were detected between groups S + V and C (Fig. 3).

#### Cytokine and immune markers production

**Fig. 4:**
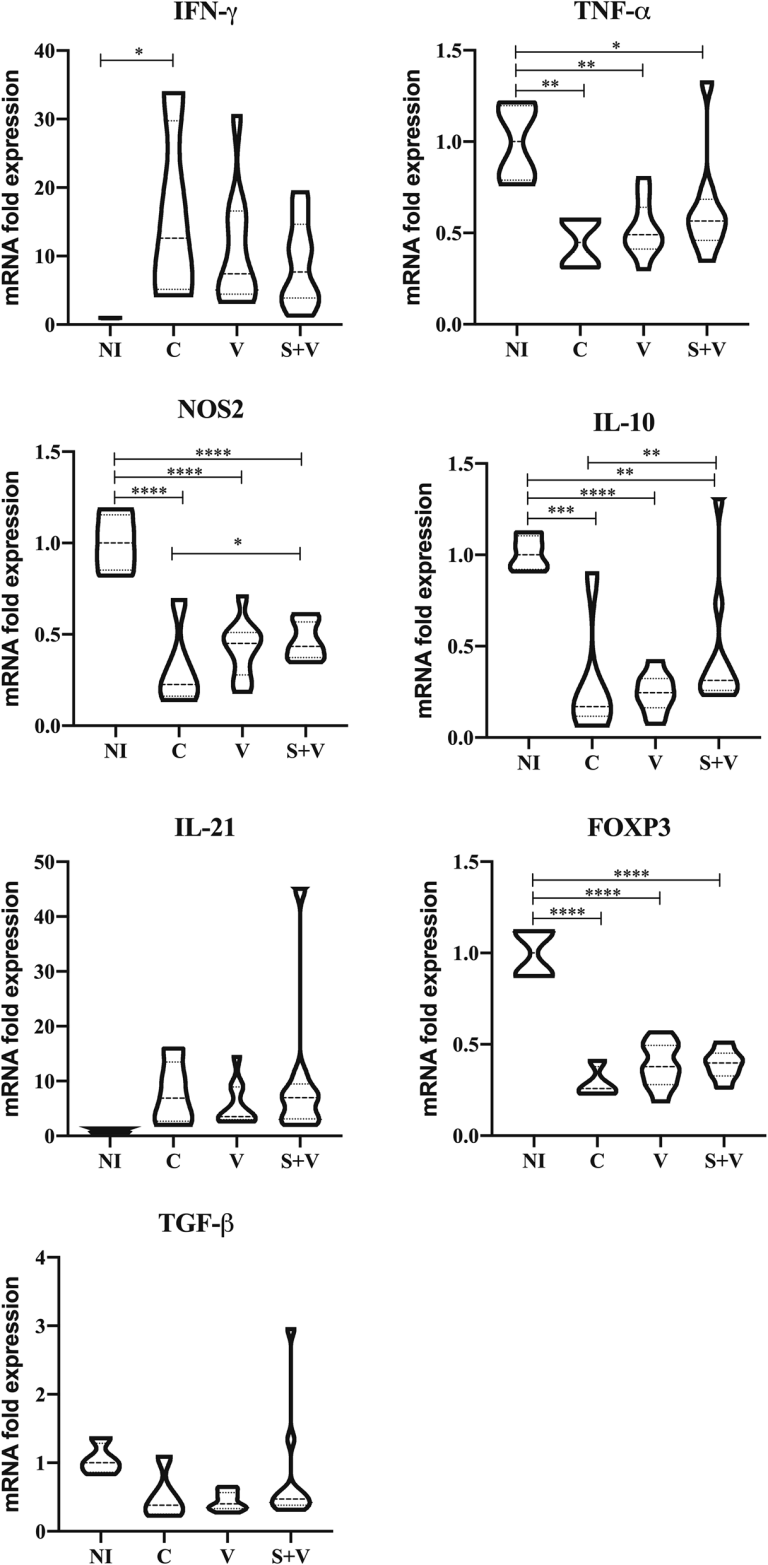
Cytokine expression in spleen samples at the end of the study. IFN-γ, TNF-α, NOS2, IL-10, IL-21, FOXP3, and TGF-β expression was determined by RT-qPCR using SYBR Green as the detection chemistry. NI: non-infected group (*n* = 4); C: PBS control group (*n* = 10); V: DNA vaccinated group (*n* = 10); S + V: sirolimus-treated and DNA vaccinated group (*n* = 10). NI group was taken as a reference group, with a value of 1. ANOVA; *P < 0.05, **P < 0.01, ***P < 0.001**** *P* < 0.0001

Expression of cytokines (IFN-γ, TNF-α, IL-10, IL-21 and TGF-β) plus the Treg immune marker FoxP3, and the NOS2 enzyme was studied and detected by the end of the study in spleen samples of all experimental groups (*n* = 34). The results are shown in Fig. 4.

First, we compared the non-infected hamsters (NI group) *versus* the PBS control infected animals (group C). *Leishmania infantum* infection (group C) significantly increased the expression of IFN-γ mRNAs ($\hat{\uppsi }$ = 80.75, 95% CI: 11.03–152.33, *P* = 0.0400), whereas it significantly reduced the expression of TNF-α ($\hat{\uppsi }$ = − 842.43, 95% CI: − 1466.92–217.95, *P* = 0.0041), IL-10 ($\hat{\uppsi }$ = 321.38, 95% CI: 125.36–551.69, *P* = 0.0080), FoxP3 ($\hat{\uppsi }$ = − 0.34, 95% CI: − 0.47–0.19, *P* < 0.0001), and NOS2 ($\hat{\uppsi }$ = − 1.04, 95% CI: − 1.74–0.38, *P* < 0.0001) mRNAs in the spleen (Fig. 4). The transcription profile of V and S + V groups were similar to that of group C when they were compared to NI animals. IFN-γ was the only exception with levels in vaccinated animals not statistically different from those of the NI group.

Both vaccinated groups (V and S + V) expressed similar transcription patterns for the studied molecules with higher expression levels of NOS2, IL-10, FoxP3, TGF-β and TNF-α, with a concurrent reduction of IFN-γ when compared to group C (Fig. 4). Statistical analysis showed that the S + V group expressed higher NOS2 (2.12-fold increase; $\hat{\uppsi }$ = − 1.04, 95% CI: − 1.74–0.38, *P* = 0.016) and IL-10 levels (1.8-fold increase; $\hat{\uppsi }$ = − 0.87, 95% CI: − 1.33–0.42, *P* = 0.010) compared with group C. The DNA vaccine alone induced an increase in NOS2 and IL-10 expression, although not significant, when compared with group C animals. No differences in TNF-α, TGF-β and FoxP3 expression were found between any vaccinated group and the group C hamsters in the spleen. There were trends ($\hat{\uppsi }$ = − 1.04, 95% CI: − 2.51–0.43, *P* = 0.0552) for both vaccinated groups (V and S + V) in reducing IFN-γ expression (0.588- and 0.609-fold, respectively) compared to group C (Fig. 4).

## Discussion

Previous studies have highlighted the need for finding new boosting strategies to potentiate protection induced by DNA immunization. Although mTOR inhibitors have been used for enhancing protection against some bacterial and viral infections, and very recently in the treatment of cutaneous leishmaniasis [30], nothing is known about their effect in a vaccination protocol against VL. To the best of our knowledge, the present study represents the first use of the immunosuppressive drug SIR in a vaccination strategy against VL with the aim of improving the immune response against this parasitic infection.

Naked DNA vaccination reduced *L. infantum* parasite loads in the lymph nodes and skin. Our results show that administering SIR during immunization along with the naked DNA vaccine significantly reduces the parasite load even in the spleen, the main *Leishmania* target organ, indicating that SIR treatment can enhance the protection provided by the naked DNA vaccine itself. Recently, the utilization of SIR as a treatment for *L. major* cutaneous leishmaniasis in mice led to a significant reduction of parasite burden in draining lymph nodes [30], also confirming that the use of mTOR inhibitors could be a new strategy to limit the growth of different *Leishmania* species.

Administering SIR during vaccination results in acceleration of the CD8+ T cell differentiation program towards memory cells [24], thus promoting enhanced specific immune responses, such as IFN-γ production. Due to the lack of reagents available for the hamster model, analysis of isolated CD8+ T cells was not possible. Expression analyses of some relevant cytokines involved in VL control and pathogenesis in spleen tissue were performed to characterize the immune response triggered in this organ after challenge. Unfortunately, immune response in other target organs was not evaluated in the present study and future experiments will show if parasite reduction in different tissues caused by SIR and DNA vaccine depends on the same immune mechanisms as in the spleen. The protection observed in S + V animals was associated with a significant increase in NOS2 expression in the spleen, an indication of adequate anti-parasite effector function. In accordance with our results, some studies have shown that inhibition of mTORC1 by rapamycin in macrophages activated the M1 phenotype, characterized by the secretion of proinflammatory cytokines, effective parasite killing, and increased expression of the NOS2 enzyme [42–44]. Levels of NO production and NOS2 expression in hamster macrophages have been associated with *Leishmania* vaccine protection in other studies [45, 46]. Despite the relative unresponsiveness of NOS2 transcription to IFN-γ in hamsters [47], other cytokines, such as TNF-α, might induce NO production in macrophages [48]. However, we did not detect a significant increase in TNF-α expression in the S + V group, though IL-1, IFN-α, or IFN-β could also be implicated in NOS2 expression.

Surprisingly, despite the parasitological protection observed in the spleen in SIR-treated hamsters, we found a decreasing trend in IFN-γ production in the same tissue, suggesting that the mediated protection in our model may not be due to the expected SIR effect (increase of IFN-γ production), or at least we could not detect it by analyzing the whole spleen at five weeks post-infection. Analysis of immune response before challenge would have been very useful to characterize the SIR and vaccination effect and future studies will address this important question. Another explanation for the unexpected decrease in IFN-γ production could be that mTORC1 inhibition enhances CD8+ T cell memory development but diminishes CD8+ T cell effector function with reduced IFN-γ production [49]. SIR administration against *L. major* infection induced enhanced specific IFN-γ production, but the treatment schedule and SIR dose were different than those used in the present study [30]. Although IFN-γ is a cytokine widely accepted to be involved in *Leishmania* control, it is also known to steadily increase during early stages of infection without a clear contribution to parasite control [47, 50]. In this line, our control infected animals showed higher levels of this cytokine when compared to non-infected controls. Moreover, recent studies have also shown that IFN-γ has a paradoxical effect in promoting *Leishmania* growth in macrophages from chronically infected hamsters [51]. Consistently, in our study, vaccinated animals presented lower IFN-γ expression levels in spleen samples than in control hamsters, suggesting that the combined activity of sirolimus with our DNA vaccine regulated the overexpression of IFN-γ, and possibly its counterproductive effect. Further studies are needed to investigate the cell source of IFN-γ and its role during disease in *L. infantum* infection. In addition, measurement of the expression of other important cytokines for *Leishmania* control, such as IL-12, could contribute to a better understanding of the protective effect generated by our vaccine protocol.

Parasitological protection in the spleen of SIR-vaccinated animals took place even under high expression levels of IL-10. A key role in the pathogenesis of murine and human VL is usually assigned to IL-10 [52, 53]. However, it is worth noting that other authors have shown that IL-10 signaling neither influences Th1 responses nor is responsible for vaccine failure in a *L. major* mouse model [54]. Conversely, it may play a pivotal role by limiting excess inflammation during infection, as suggested for other intracellular pathogens [55, 56]. In our model, *L. infantum* infection (group C) caused a significant decrease of this cytokine production with regard to the non-infected controls (group NI); in contrast, vaccinated animals (V/S + V) showed a slight recovery of IL-10 levels. Other vaccine attempts with *L. infantum* antigens in dogs have also shown mixed pro- and anti-inflammatory cytokine environments, with higher IL-10 production induced in vaccinated animals [57, 58]. These results suggest that IL-10 might play a beneficial immune regulatory role in *L. infantum* infection.

Other immunoregulatory markers such as IL-21, FoxP3 and TGF-β showed no significant expression variations between vaccinated and infected control group (group C) although a tendency of being dysregulated during infection was detected. In our study, the FoxP3 marker was downregulated during infection and presented a tendency to increase in the S + V group. FoxP3+ regulatory T cells (Tregs) produce TGF-beta and IL-10 but their role in this parasitic disease is still not clear [53]. In this regard, IL-21 has an important role amplifying IL-10 production by type 1 regulatory T cells (Tr1) in VL patients [59]. However, in our study, *L. infantum-*infected hamsters expressed high levels of IL-21 cytokine that did not seem correlated with the changes observed in IL-10 expression. More information is needed to elucidate the actual role of Treg and Tr1 cells in VL protection, which is becoming essential to the understanding which cells are responsible for the observed differences.

Plasmid DNA vaccines are also known to promote antibody production [16, 60]. However, serology levels in our study revealed a significant decrease of anti-*Leishmania*-specific IgG antibodies five weeks post-challenge in hamsters receiving the vaccine alone when compared to the group C. These reduced antibody levels have already been observed in a previous experimental trial of this vaccine and may be explained by the capacity of the vaccine to control the aberrant B cell differentiation widely associated with disease progression [31]. Surprisingly, adding SIR to the immunization protocol translated into a significant increase in *Leishmania*-specific IgG levels compared to animals receiving the DNA vaccine alone, which were close to the control group. An association between increased antibody responses due to polyclonal B cell activation, elevated parasite burden, and the absence of proliferative responses, has been described in the VL hamster model [61]. However, the increase in *Leishmania*-specific IgG levels in animals treated with SIR observed in our study was not accompanied by an increase in parasite burden. SIR administration during an immunization assay with influenza virus did not alter specific IgG antibody levels compared to infected non-protected controls [25]. Detection of specific antibodies against each one of the antigen vaccine candidates would be needed to further characterize the SIR response.

## Conclusions

The co-administration of SIR potentiated the protection conferred by the DNA vaccine carrying the *Leishmania* genes *TRYP*, *PAPLE22*, *KMPII* and *LACK* in the pVAX vector assayed in this study. The protection achieved was associated with enhanced NOS2 expression accompanied by high IL-10 mRNA levels. The role of mTORC1 is complex and immune cell type-dependent [62], therefore, further studies are needed to characterize its role in T-cells and macrophages during *L. infantum* vaccination.

## Data Availability

All data generated or analyzed during this study are included in this published article.

## Abbreviations

VL: visceral leishmaniasis
SIR: sirolimus
mTOR: mechanistic target of rapamycin
RPL18: ribosomal protein L18
IFN-γ: interferon γ
FoxP3: fork head box P3
NOS2: nitric oxide synthase 2
TGF-β: transforming growth factor β
TNF-α: tumor necrosis factor α
IL: interleukin
IgG: immunoglobulin G
KMPII: kinetoplastid membrane protein-11
LACK: *Leishmania* homologue of receptors for activated C kinase
PAPLE22: potentially aggravating protein of *L. infantum*
TRYP: tryparedoxin peroxidase
CTLA: crude total *Leishmania* antigen
qPCR: quantitative polymerase chain reaction
F: forward primer
R: reverse primer
P: probe
